# Mathematical indices for the influence of risk factors on the lethality of a disease

**DOI:** 10.1007/s00285-021-01700-4

**Published:** 2021-12-08

**Authors:** Ricardo Martínez, Joaquín Sánchez-Soriano

**Affiliations:** 1 Departmento de Teoría e Historia Económica, Universidad de Granada, Granada, Spain; 2 I.U. Centro de Investigación Operativa (CIO), Universidad Miguel Hernández de Elche, Elche, Spain

**Keywords:** Epidemiology, Comorbidity, Lethality, Equal attribution index, Shapley value index, COVID-19, 90B99, 91A12, 91A40, 91A80, 91B82, 92D30

## Abstract

We develop a theoretical model to measure the relative relevance of different pathologies of the lethality of a disease in society. This approach allows a ranking of diseases to be determined, which can assist in establishing priorities for vaccination campaigns or prevention strategies. Among all possible measurements, we identify three families of rules that satisfy a combination of relevant properties: *neutrality*, *irrelevance*, and one of three *composition* concepts. One of these families includes, for instance, the Shapley value of the associated cooperative game. The other two families also include simple and intuitive indices. As an illustration, we measure the relative relevance of several pathologies in lethality due to COVID-19.

## Introduction

The COVID-19 pandemic is exerting a substantial global impact both from a humanitarian and economic perspective. As of February 2021, the virus has already resulted in over two million deaths. As with many other diseases, it is crucial to determine how preexisting conditions (e.g., other pathologies or genetic factors) influence the evolution and end of the disease. Identifying risk factors is relevant for the design of efficient public health systems and health services, including strategic interventions that can limit the fatal outcomes of a disease. However, when several factors are present and when these do not always occur simultaneously, the following question reasonably arises: which factors are the most relevant to a prognosis? The ability to quantify the relevance of a pathology in the observed mortality is a determinant in the design of a successful strategy to combat a disease. This is especially the case when establishing priorities between different population groups in the design of a vaccination campaign. In the case of COVID-19, several papers have examined the influence of preexisting comorbidities on COVID-19 mortality. For example, using logistic regression, Nogueira et al. (2020) evaluated and ranked the risk factors for COVID-19 mortality in Portugal. In the New York City Area, Richardson et al. (2020) studied risk factors for the evolution of hospitalized patients with COVID-19. Bello-Chavolla et al. (2020) used Cox proportional-hazards regression to score risk factors of lethality in patients with COVID-19 in Mexico. Stoian et al. (2020) statistically analyzed patterns of comorbidity, gender, and age in the mortality of patients with COVID-19 in Romania. Therefore, it is of research interest to examine the extent to which each risk factor contributes to COVID-19 mortality.

A major aim of epidemiological research is to measure disease occurrence in relation to specific variables, which are known as risk factors. Impact measures are used to assess the contribution of one or several risk factors to the occurrence of incident cases at the population level (Benichou 2007). Therefore, in a given population, the methodological task of adequately evaluating the impact of risk factors, which are not always present at the same time, on the final outcome of a disease is a relevant mathematical question. The first major problem involves determining how to assess risk factors that contribute to a particular goal. Since we can view risk factors as collaborating to achieve a goal, one possibility is to approach the problem from the perspective of cooperative game theory. In this case, it is first necessary to associate a cooperative game with the problem at hand, and then to apply a solution concept. One of the most commonly used solutions is the Shapley value (Shapley 1953). Its widespread use is due to its relevant properties and simple interpretation (see, for instance, Roth 1988; Algaba et al. 2019b). Another alternative is to use the theory of distributive justice (Rawls 1971; Roemer 1996) to define indices or measures that are closely related to the analyzed attribution problem, and which have properties that make them relevant and suitable in the corresponding context. On many occasions, the approaches are interrelated, studying whether the solutions provided in one of the approaches correspond to some concept or principle of the other. Moreover, these approaches can be also carried out in other biological systems in which several factors are implicated in producing a desirable or non-desirable result (e.g., climate change, genetics, or artificial and biological networks).

The most popular measures of epidemiological risk are relative risk, the odds rate, and attributable risk (Levin 1953). On this last concept of risk measure, we can find different approaches from the perspective of game theory when there are several risk factors. Cox (1985) investigated the problem of risk attribution by considering a risk function from the set of risk factors to [0, 1]. This can be seen as the characteristic function of a game, where the factors play the role of the players and the risk allocation functions are defined. In turn, the author demonstrated that there exists a unique risk allocation function that satisfies three reasonable properties in the epidemiological context: *additivity*, *independence of labeling*, and *independence of irrelevant factors*. Moreover, this risk allocation function is the Shapley value of the risk attribution problem seen as a game. Eide and Gefeller (1995) and Gefeller (1994) introduced sequential and average attributable fractions as measures of risk based on the results of Cox (1985), particularly those related to the Shapley value. Land and Gefeller (1997) and Gefeller et al. (1998) provided a game theoretic justification of the results in Eide and Gefeller (1995) by means of a set of axioms (*symmetry*, *marginal rationality*, and *internal marginal rationality*) different from those used in Cox (1985). Likewise, they used the (multifactorial) attributable risk function instead of a general risk function. McElduff et al. (2002) and Llorca and Delgado-Rodriguez (2004) introduced a proportional weighting scheme to distribute attributable risk among the different risk factors, and Rabe and Gefeller (2006) compared this method to the one based on the Shapley value. To finish with the problem of distributing attributable risk, Land and Gefeller (1998, 2000) introduced a multiplicative version of the Shapley value to distribute the attributable risk.

Cooperative games have been also applied to attribution problems arising from biological situations, particularly from genetics. For example, Moretti et al. (2007) introduced microarray games to analyze the relevance of genes. The authors proposed the Shapley value of the game as a relevance index for genes based on properties with a genetic interpretation (*partnership rationality*, *partnership feasibility*, *partnership monotonicity*, *equal splitting*, *null gene*). Lucchetti et al. (2010) investigated the Shapley value and the Banzhaf value (Banzhaf 1965) for microarray games by considering new relevant properties: *symmetry*, *individual consistency*, *average loss*, and *total loss*. Moretti et al. (2010) and Cesari et al. (2018) analyzed the co-expression networks of genes to explore the relevance of genes in terms of their relationships with other genes, relying on centrality indices in networks and the Shapley value of an associated game. Microarray games were also applied to study neuroblastic tumors in Albino et al. (2008) and to detect the genes involved in autism in Esteban and Wall (2011).

Phylogenetic trees are arborescent schemes that illustrate evolutionary relationships between various species or other entities that are believed to have a common ancestry. These schemes are useful for measuring (genetic) biodiversity. Therefore, if we are interested in preserving biodiversity, the problem of finding adequate measures to quantify the biological diversity of a species or genus is of great biological interest. In one sense, this is an attribution problem where we are interested in knowing which species are responsible for what part of the diversity. For each (unrooted) phylogenetic tree, Haake et al. (2008) defined a game (known as a phylogenetic tree game) and proposed the Shapley value of that game as a suitable measure of the diversity of species. Moreover, the authors characterized the Shapley value of those games by means of a set of axioms that are considered relevant for a diversity measure: *Pareto efficiency*, *symmetry*, *additivity*, and *group proportionality*. Redding et al. (2008) demonstrated that the Shapley value in Haake et al. (2008) and the fair proportion index (Redding and Mooers 2006) are highly correlated. Also, Hartmann (2013) showed that the Shapley value and the fair proportion index become equivalent when the number of species increases. However, Fuchs and Jin (2015) proved that the Shapley value of the (rooted) phylogenetic tree game and the fair proportion index are, in fact, the same, and Fuchs and Paningbatan (2020) studied the correlation between the (unrooted) Shapley value and the fair proportion index when the $\beta$-splitting model is used to generate random phylogenetic trees. More recently, Stahn (2020) studied the main differences between the Shapley values of phylogenetic tree games, and Wicke and Steel (2020) examined the combinatorial properties of phylogenetic diversity index, including the different versions of the Shapley value.

As mentioned above, attribution problems are relevant in many other fields. For example, Brander et al. (2011) offered an account of the importance of the attribution problem in climate change, and Burger et al. (2020) conducted an in-depth review of the attribution problem in the context of climate change, both from a technical perspective and its legal and policy applications. A final example of attribution problems is in the field of artificial and biological networks. In this case, the attribution problem refers to measuring the contribution of each element of a network to a function for the successful performance of that function. Keinan et al. (2004) used the Shapley value for fair attribution of functional contribution in networks and provided a wide range of potential applications of this approach.

In this paper, we approach the epidemiological problem of multifactorial risk attribution, but we directly use the risk profile of individuals in the population, as in the microarray problems in Moretti et al. (2007) and others. This is in contrast to using attributable risk, as in Cox (1985), Eide and Gefeller (1995), or Gefeller (1994). Moreover, instead of considering solutions for an associated game, we directly consider population data to define indices for the influence of risk factors on the lethality of a disease. Another difference compared to previous approaches is that we only consider individuals who have a specific outcome in the development of the disease and not all possible outcomes. Therefore, we measure the relative influence of risk factors in a particular outcome (e.g., the lethality of a disease). In this way, we obtain a ranking of the influence of risk factors on the outcome of interest (i.e., seeking to identify the risk factors that have the greatest impact on the outcome of interest).

To be more precise, in our setting, a problem is determined by a set of pathologies, a set of individuals who have passed away, and a lethality matrix, which specifies the pathologies that led to an individual’s death. An index is a measure of the lethality relevance of the pathologies as a function of the lethality matrix. Following the axiomatic methodology in the theory of fair distribution (Rawls 1971; Roemer 1996), we investigate whether there exist indices that satisfy combinations of properties which are suitable in this context. In particular, we focus on three axioms: *neutrality*, *irrelevance*, and *composition*. The first of these says that the mere name of the pathology should not affect the measurement. *Irrelevance* states that the relevance lethality of an irrelevant pathology is zero. Finally, *composition* requires that when we bring together data from two subgroups, the index of the total group can be determined by a suitable composition of the indices of the subgroups. We consider three types of composition: *additive composition*, *sized composition*, and *incidence composition*. *Additive composition* requires that the index is additive with respect to the set of individuals; *sized composition* requires that the index is weighted additive with respect to the size of the subgroups of individuals; and *incidence composition* requires that the index is weighted additive with respect to the incidence of pathologies in each subgroup. The combination of *neutrality*, *irrelevance*, and one of the composition properties give rise to a unique family of indices for the influence of risk factors on the lethality of a disease. In this way, we obtain three families of indices. Each of these families has as a member a simple index that is remarkable and easy to interpret. In particular, the family obtained with *additive composition* contains the *equal attribution index*, which emerges as the most convenient alternative of that family since it coincides with the Shapley value of the natural associated game. The family obtained with *sized composition* contains the *share index*, which measures the proportions of individuals who died with each pathology. Finally, the family obtained with *incidence composition* contains the *ratio index*, which measures the ratio of pathologies out of all possible cases.

There are several problems in the literature consisting of a set of attributes and a population whose individuals have at least one of those attributes (see Algaba et al. 2019a). Then, a game is associated with each problem, and the measure of relevance of each attribute usually coincides with a solution concept of that cooperative game. Efficiency is one of the basic requirements for the solution. One of these problems is the museum pass problem (Ginsburgh and Zang 2003). Our model also presents several particularities with respect to this problem and those related to it. First, we do not require efficiency in the definition of the lethality influence index. The lethality relevances do not need to add up to a given amount, and different indices may add up differently. And second, we consider the possibility that there are individuals in the population who do not have any of the attributes, that is, there may be individuals who have died without any of the considered pathologies. This possibility is explicitly excluded in Ginsburgh and Zang (2003), Bergantiños and Moreno-Ternero (2015), and Dehez and Ginsburgh (2020). In this sense, our results, if properly applied to each domain, can be also understood as a generalization of these papers, when we eliminate efficiency. Indeed, one of the families of indices we characterize contains the solution proposed by these authors for their frameworks.

Finally, to illustrate the application of our theoretical framework, we measure the relevance of several pathologies in the lethality of COVID-19. According to the Novel Coronavirus Pneumonia Emergency Response Epidemiology Team (see Team 2020), the most prominent comorbidities implicated in COVID-19 mortality are hypertension, cardiovascular disease, diabetes, and chronic respiratory disease. From their data, we run several simulations to apply the indices we characterize. We find that the relevance of some pathologies differs from the impact reported in their study.

The rest of the paper is organized as follows. Section 2 presents the mathematical model we will use. In Sect. 3, we propose some simple and intuitive indices that are suitable for measuring the relative relevance of pathologies of the lethality in society, one of which is the equal attribution index. In Sect. 4, we present some properties that we consider relevant in this context. Our main results are given in Sect. 5. We characterize the three families of rules that satisfy the properties. The equal attribution index belongs to one of these families and we show that it coincides with the Shapley value of an associated game. Section 6 illustrates the application of the equal attribution index of lethality relevance to the case of COVID-19. Finally, Sect. 7 offers concluding remarks.

## Mathematical model of comorbidity in the lethality of a disease

Let $P=\{1,\ldots ,p\}$ be a set of **pathologies**. Suppose we want to assess their relevance as the cause of death in a group of **individuals** $N=\{1,\ldots , n\}$. A **lethality matrix** is a matrix $X \in \{0,1\}^{n \times p}$ of *n* rows (one for each individual) and *p* columns (one for each pathology), where

$$\begin{aligned} x_{ia}= {\left\{ \begin{array}{ll} 1 &{} \text {if { i} dies with pathology { a}}, \\ 0 &{} \text {otherwise}. \end{array}\right. } \end{aligned}$$

We denote by $x_{i \cdot }$ the *i*-th row of *X*, which indicates the pathologies that *i* has. We also denote by $x_{\cdot a}$ the *a*-th column of *X*, which indicates the individuals with pathology *a*. Let $\mathcal {D}^{N}$ be the domain of all possible matrices with individuals in *N*.

We shall also consider a variable-population generalization of the model. Then, there is a set of potential individuals, which are indexed by the natural numbers $\mathbb {N}$. Let $\mathcal {N}$ be the set of finite subsets of $\mathbb {N}$, with generic element *N*. We denote by $\mathcal {D} \equiv \bigcup _{N\in \mathcal {N}}\mathcal {D}^{N}$ the class of all possible matrices with variable population.

Although we have only mentioned pathologies in the model, it is possible to consider other risk factors such as gender and age without the need to make any special modifications to the model. Therefore, the model is sufficiently general to analyze other types of biological problems in which the impact of different factors must be assessed.

Lethality relevance is measured using an **index**. It is a mapping $\lambda : \mathcal {D} \longrightarrow \mathbb {R}^p_{\ge 0}$ that assigns a vector $\lambda (X)$ to each lethality matrix $X \in \mathcal {D}^N$, where $\lambda _a(X)$ is the relevance of pathology $a \in P$ on the lethality.

### Example 1

Consider the case of a group of individuals $N=\{1,\ldots ,6\}$ who die with one or several pathologies in $P=\{\text {diabetes}, \text {high blood pressure}, \text {bronchitis}\}$. Data are represented using a lethality matrix as follows:

$$\begin{aligned} X = \left( \begin{array}{ccc} 0 &{}\quad 1 &{}\quad 1 \\ 0 &{}\quad 0 &{}\quad 0 \\ 0 &{} \quad 1 &{} \quad 1 \\ 1 &{}\quad 0 &{}\quad 0 \\ 1 &{}\quad 1 &{}\quad 1 \\ 1 &{}\quad 0 &{}\quad 1 \\ \end{array} \right) . \end{aligned}$$

The first row indicates that Agent 1 died with high blood pressure and bronchitis but was not diabetic. We observe that three out of six people had diabetes when they passed away, the same number as those with high blood pressure. Also, in this example, any individual with high blood pressure was affected by bronchitis. At this point, the obvious question is: what is the relevance of each pathology on the lethality of this society?

## Lethality influence indices

This section presents several lethality influence indices that can be applied to quantify the relevance of risk factors (preexisting pathologies) on the lethality of a disease.

The first lethality influence index, relevance, is relatively straightforward. Relevance is defined as the number of individuals who died having been diagnosed with the pathology. Just counting the number of cases (and obviating the size of the population, for example) may not be very accurate, but it can be considered as a primary measure in epidemiology. In fact, this indicator has been recurrent in the media during the pandemic.

**Count index**. For each $X \in \mathcal {D}^N$ and each $a \in P$,

$$\begin{aligned} \lambda ^C_a(X) = \sum _{i=1}^n x_{ia}. \end{aligned}$$

The next index states that lethality is the share of individuals who died having been diagnosed with a pathology. The objective of this index is to measure what proportion of the deceased individuals had a certain pathology. This kind of index can also be considered as a primary measure in epidemiology and, in addition, it is common to find it in epidemiological reports and studies (see, for example, Sanyaolu et al. 2020).

**Share index**. For each $X \in \mathcal {D}^N$ and each $a \in P$,

$$\begin{aligned} \lambda ^S_a(X) = \frac{1}{n} \sum _{i=1}^n x_{ia}. \end{aligned}$$

The third alternative measures lethality as the share of occurrences of a pathology out of all possible cases. While the previous index only takes into account what proportion of individuals suffered from a certain pathology, this measure collects the effect that individuals may have more than one pathology and, therefore, what it measures is the impact on the total number of pathologies suffered by the deceased. For example, if a certain pathology were present in all individuals, its share index would be 1, but if all the deceased had, in addition, two other different pathologies each, this should be taken into account when evaluating its influence on lethality because it was always accompanied by two other pathologies. In this example, its ratio index would be 0.33, which better captures this circumstance.

**Ratio index**. For each $X \in \mathcal {D}^N$ and each $a \in P$,

$$\begin{aligned} \lambda ^R_a(X) = {\left\{ \begin{array}{ll} \displaystyle {\frac{1}{\Vert X\Vert _1} \sum _{i=1}^n x_{ia}} &{} \text {if } \Vert X\Vert _1 > 0 \\ [0.3cm] 0 &{} \text {if } \Vert X\Vert _1 =0 \end{array}\right. }, \end{aligned}$$

where $$\Vert X\Vert _1 = \sum _{a \in P}\sum _{i=1}^{n}x_{ia}$$.^1^

The last index is also simple. It works as follows: assign to each of the deceased a mass of 1 unit; then, split this mass among the pathologies the individual experienced; finally, add across individuals. At this point it is legitimate to wonder why the mass of 1 unit should be equally split among the different pathologies. We must take into account that the mass 1 corresponds to one individual who died with several pathologies. Ex ante, it may not be possible to know the primary cause of the death of such an individual, especially if it is evaluated in conjunction with a disease that is common to all cases (COVID-19, for instance). When it is not feasible to explore in depth the causes of death of each single person, it is natural and convenient to assume that all pathologies are equally responsible for the decease of that individual. Applying the same reasoning to all individuals and combining the data, it is possible to capture the relative relevance of each pathologies in the whole population. That is the goal of the *equal attribution index*.

**Equal attribution index**. For each $X \in \mathcal {D}^N$ and each $a \in P$,

$$\begin{aligned} \lambda ^E_a(X) = \displaystyle {\sum _{i \in N_a^{*}} \frac{x_{ia}}{\Vert x_{i \cdot } \Vert _1}}, \end{aligned}$$

where $$N_a^{*}=\{i \in N: x_{ia} = 1 \}$$.

### Example 2

Continuing with Example 1, we first illustrate the functioning of the equal attribution index. From matrix *X*, we construct the following matrix:

$$\begin{aligned} X^E = \left( \begin{array}{ccc} 0 &{}\quad \frac{1}{2} &{}\quad \frac{1}{2} \\ 0 &{} \quad 0 &{}\quad 0 \\ 0 &{}\quad \frac{1}{2} &{}\quad \frac{1}{2} \\ 1 &{}\quad 0 &{}\quad 0 \\ \frac{1}{3} &{}\quad \frac{1}{3} &{}\quad \frac{1}{3} \\ \frac{1}{2} &{}\quad 0 &{}\quad \frac{1}{2} \\ \end{array} \right) . \end{aligned}$$

Each row has a mass of one unit. Individual 1, represented by the first row, has diabetes and high blood pressure, and so the mass is split between these pathologies by assigning a weight of $\frac{1}{2}$ each. The procedure is applied to the rest of the population. Finally, we sum the weights to obtain the relevance of each pathology. Thus,

$$\begin{aligned} \lambda ^E(X) = \left( 1 + \frac{1}{3} + \frac{1}{2}, \frac{1}{2} + \frac{1}{2} + \frac{1}{3}, \frac{1}{2} + \frac{1}{2} + \frac{1}{3} + \frac{1}{2}\right) = \left( \frac{11}{6}, \frac{8}{6}, \frac{11}{6} \right) . \end{aligned}$$

From the information in *X*, we conclude that diabetes and bronchitis are equally relevant in the lethality of this disease in society. Also, both diabetes and bronchitis have a greater impact compared to high blood pressure, with the significance of high blood pressure being 27.3% less than the other two pathologies.

For the same example, the count index is

$$\begin{aligned} \lambda ^C(X) = \left( 3,3,4 \right) . \end{aligned}$$

As we observe, different results are obtained with this index. According to the count index, the two diseases with the same relevance are diabetes and high blood pressure. On applying the share index, we get

$$\begin{aligned} \lambda ^S(X) = \left( \frac{1}{2}, \frac{1}{2}, \frac{2}{3} \right) . \end{aligned}$$

Notice that the count and share indices differ in absolute terms, but the relative relevance of any pair of pathologies is the same in both methods. Finally, lethality relevance according to the ratio index is

$$\begin{aligned} \lambda ^R(X) = \left( \frac{3}{10}, \frac{3}{10}, \frac{4}{10} \right) . \end{aligned}$$

Given a lethality matrix $X \in \mathcal {D}$, we define the **aggregate impact** of the pathologies in *P* as the aggregation of lethality relevances $\Lambda (X)=\sum _{a \in P} \lambda _a(X)$. It is worth noting that the aggregate impacts of different indices, as the previous example illustrates, are different in general. Indeed, in Example 2, $\Lambda ^E(X)=5$, $\Lambda ^C(X)=10$, $\Lambda ^S(X)=\frac{5}{3}$, and $\Lambda ^R(X)=1$.

## Relevant properties for a lethality influence index

The first axiom is a standard principle of impartiality. It simply requires that the name of the pathology should not be relevant for the measurement of lethality relevance.

**Neutrality**. For each $X \in \mathcal {D}^N$,

$$\begin{aligned} \lambda (\pi (X)) = \pi (\lambda (X)), \end{aligned}$$

where $\pi (X)$ is a permutation of the columns of *X* and, since we can identify columns with pathologies, $\pi (\lambda (X))$ is the same permutation applied to the vector $\lambda (X)$. Furthermore, since $\pi$ can be looked at as a bijective mapping from *P* to *P*, we abuse notation and also denote by $\pi$ the permutation of the elements of the set of pathologies *P*, where $\pi (a)$ is the new number (column in the matrix) associated with pathology *a* when permutation $\pi$ is applied.^2^

We say that a pathology is *irrelevant* for a lethality if it was not present in any death. The next property says that the lethality relevance of an irrelevant pathology must be zero.

**Irrelevance**. For each $X \in \mathcal {D}^N$ and each $a \in P$, if $x_{ia}=0$ for all $i \in N$, then $\lambda _a(X)=0$.

Suppose there are two disjoint groups of individuals *N* and *M* (e.g., two regions of a country). Now consider a larger society resulting from combining *N* and *M*. The question is how to recalculate the index for $N \cup M$ from the indices for *N* and *M*. The name of this recalculation is *composition*. Depending on how the composition of the indices for *N* and *M* is undertaken, different composition properties will be generated. *Additive composition* states that the relevance on the lethality of each pathology in the large population, $N \cup M$, is the sum of the relevance in *N* and *M*.

**Additive composition**. Let $N,M \in \mathcal {N}$ such that $N \cap M = \varnothing$. For each $X \in \mathcal {D}^N$ and each $Y \in \mathcal {D}^M$,

$$\begin{aligned} \lambda (X \oplus Y) = \lambda (X) + \lambda (Y), \end{aligned}$$

where $X \oplus Y \in \mathcal {D}^{N \cup M}$ is the matrix resulting from stacking *X* above *Y* (by rows).

However, we could consider that population size should be taken into account when relating the relevance of the pathologies in the larger population and the smaller ones. *Sized composition* states that the relationship between the relevance on the lethality of each pathology in the large population and the relevance in the populations *N* and *M* must be weighted by their sizes.

**Sized composition**. Let $N,M \in \mathcal {N}$ such that $N \cap M = \varnothing$. For each $X \in \mathcal {D}^N$ and each $Y \in \mathcal {D}^M$,

$$\begin{aligned} (n+m)\lambda (X \oplus Y) = n\lambda (X) + m\lambda (Y). \end{aligned}$$

Finally, we could consider that the incidence of pathologies in each population should be taken into account, and then a new composition property could be defined. *Incidence composition* says that the relationship between the relevance on the lethality of each pathology in the large population and the relevance in the populations *N* and *M* must be weighted by the incidence of the pathologies in each population.

**Incidence composition**. Let $N,M \in \mathcal {N}$ such that $N \cap M = \varnothing$. For each $X \in \mathcal {D}^N$ and each $Y \in \mathcal {D}^M$,

$$\begin{aligned} \Vert X\oplus Y \Vert _1\lambda (X \oplus Y) = \Vert X \Vert _1\lambda (X) + \Vert Y \Vert _1\lambda (Y). \end{aligned}$$

Next, we explore the compatibility or incompatibility of some groups of the previous properties. In this regard, Proposition 1 states that the three composition requirements are mutually excluding. More specifically, there is no lethality index, other than the null index, that satisfies any pair of compositions.

### Proposition 1

The null index, $\lambda ^0(X) =0_P$ for all $N \in \mathcal {N}$ and for each $X \in \mathcal {D}^N$, is the unique index that satisfies the following pairs of properties:

i) Additive composition and sized composition.
ii) Additive composition and incidence composition.

### Proof

Since $\lambda ^0(X) =0$ for each $X \in \mathcal {D}$, it is obvious that it satisfies the three composition properties.

Let us suppose that there exists a non-null index $\lambda$ satisfying additive composition and sized composition. Since this index is not null, there exists $X \in \mathcal {D}^N$ such that $\lambda (X) \ne 0_P$. Next we take *X* and any $Y \in \mathcal {D}^M$, $N \cap M = \varnothing$, since $\lambda$ satisfies additive composition and sized composition, then

$$\begin{aligned} n\lambda (X) + m\lambda (Y)=(n+m)\lambda (X \oplus Y)= (n+m)(\lambda (X) + \lambda (Y)) \Rightarrow m\lambda (X) + n\lambda (Y) = 0_P, \end{aligned}$$

but this is impossible.

Let us now suppose that there exists an index $\lambda$ satisfying additive and incidence composition. Since this index is not null, there exists $X \in \mathcal {D}^N$ such that $\lambda (X) \ne 0_P$. Next we take *X* and any $Y \in \mathcal {D}^M$ so that $\Vert Y\Vert _1>0$, $N \cap M = \varnothing$, since $\lambda$ satisfies additive and incidence composition, then

$$\begin{aligned} \Vert X \Vert _1\lambda (X) + \Vert Y \Vert _1\lambda (Y)= (\Vert X \Vert _1 + \Vert Y \Vert _1)(\lambda (X) + \lambda (Y)) \Rightarrow \Vert Y \Vert _1\lambda (X) + \Vert X \Vert _1\lambda (Y) = 0_P, \end{aligned}$$

but this is again impossible. $\square$

### Proposition 2

Constant indices, $\lambda ^K(X) =K, K \in \mathbb {R}^P_{\ge 0}$ for each $X \in \mathcal {D}$, are the unique indices that satisfies sized composition and incidence composition.

### Proof

It is straightforward to prove that constant indices satisfy sized composition and incidence composition. Let $\lambda$ be an index that satisfies sized and incidence composition, then we have that

1. For each $X \in \mathcal {D}^N$ and for each $Y \in \mathcal {D}^M$, so that $(m\Vert X \Vert _1 - n\Vert Y \Vert _1) \ne 0$, $\Vert Y \Vert _1 >0$, $N \cap M = \varnothing$, it holds that
  $$\begin{aligned} \begin{array}{ll} \Vert X \Vert _1\lambda (X) + \Vert Y \Vert _1\lambda (Y)= (\Vert X \Vert _1 + \Vert Y \Vert _1)(\frac{n}{n+m}\lambda (X) + \frac{m}{n+m}\lambda (Y)) &{} \\ [0.3cm] \Rightarrow m\Vert X \Vert _1\lambda (X) + n\Vert Y \Vert _1\lambda (Y)=n\Vert Y \Vert _1\lambda (X) + m\Vert X \Vert _1\lambda (Y)&{}\\ [0.3cm] \Rightarrow (m\Vert X \Vert _1 - n\Vert Y \Vert _1)\lambda (X) = (m\Vert X \Vert _1 - n\Vert Y \Vert _1)\lambda (Y). &{} \end{array} \end{aligned}$$
  Therefore, $$\lambda (X) = \lambda (Y)$$.
2. For each $X \in \mathcal {D}^N$ and for each $Y \in \mathcal {D}^M$, so that $(m\Vert X \Vert _1 - n\Vert Y \Vert _1) = 0$, $\Vert Y \Vert _1 >0$, $N \cap M = \varnothing$, we can take $Y' \in \mathcal {D}^{M'}$ with $m' = m$, $N \cap M' = \varnothing$ and $M \cap M' = \varnothing$, and such that $\Vert Y \Vert _1 \ne \Vert Y' \Vert _1 >0$. We can now apply the previous case and obtain that $\lambda (X) = \lambda (Y')$ and $\lambda (Y) = \lambda (Y')$, therefore $\lambda (X) = \lambda (Y)$.
3. For each $X \in \mathcal {D}^N$ with $\Vert X \Vert _1=0$ and for each $Y \in \mathcal {D}^M$ with $\Vert Y \Vert _1 =0$, we can do the same as in the previous case and also obtain that $\lambda (X) = \lambda (Y)$.

Now, let $X \in \mathcal {D}^N$ and $Y \in \mathcal {D}^M$ be two lethality matrices, and let $Z \in \mathcal {D}^Q$ be a lethality matrix such that $N \cap Q = \varnothing$ and $M \cap Q = \varnothing$. The pairs of lethality matrices *X*, *Z* and *Y*, *Z* will be in the conditions of one of the three cases described above. Therefore, we have that $\lambda (X) = \lambda (Z)$ and $\lambda (Y) = \lambda (Z)$, so we conclude that $\lambda (X) = \lambda (Y)$.

Therefore, if an index $\lambda$ satisfies sized and incidence composition then $\lambda (X) = K$ for all $X \in \mathcal {D}$, for some $K \in \mathbb {R}^P_{\ge 0}$. $\square$

Proposition 1 and Proposition 2 imply that any group of compatible properties (which do not lead to a constant index) has, at most, three requirements, namely neutrality, irrelevance, and one of the composition. Proposition 3 shows that any of the compositions is compatible with both neutrality and irrelevance. More precisely, it states that there exist indices that satisfy any pair of those three requirements but violate the third one.

### Proposition 3

There are non-null indices that satisfy exactly two properties and violate the remaining.

### Proof

i) The $0-1$ index satisfies neutrality and irrelevance, but it violates the three composition properties. For each $X \in \mathcal {D}^N$ and each $a \in P$,
  $$\begin{aligned} \lambda ^{0-1}_a(X) = {\left\{ \begin{array}{ll} 0 &{} \text {if } a \text { is irrelevant}, \\ 1 &{} \text {if } a \text { is not irrelevant}. \end{array}\right. } \end{aligned}$$
ii) The following index satisfies neutrality and additive composition, but it violates irrelevance. For each $X \in \mathcal {D}^N$ and each $a \in P$,
  $$\begin{aligned} \lambda ^1_a(X) = \Vert X\Vert _1. \end{aligned}$$
iii) The following index satisfies irrelevance and additive composition, but it violates neutrality. For each $X \in \mathcal {D}^N$ and each $a \in P$,
  $$\begin{aligned} \lambda ^2_a(X) = {\left\{ \begin{array}{ll} \displaystyle {\sum _{i=1}^n x_{ia}} &{} \text {if } a=1, \\ [0.3cm] 0 &{} \text {otherwise}. \end{array}\right. } \end{aligned}$$
iv) The following index satisfies neutrality and sized composition, but it violates irrelevance. For each $X \in \mathcal {D}^N$ and each $a \in P$,
  $$\begin{aligned} \lambda ^3_a(X) = \frac{1}{n}\Vert X\Vert _1. \end{aligned}$$
v) The following index satisfies irrelevance and sized composition, but it violates neutrality. For each $X \in \mathcal {D}^N$ and each $a \in P$,
  $$\begin{aligned} \lambda ^4_a(X) = {\left\{ \begin{array}{ll} \displaystyle {\frac{1}{n}\sum _{i=1}^n x_{ia}} &{} \text {if } a=1, \\ [0.3cm] 0 &{} \text {otherwise}. \end{array}\right. } \end{aligned}$$
vi) The following index satisfies neutrality and incidence composition, but it violates irrelevance. For each $X \in \mathcal {D}^N$ and each $a \in P$,
  $$\begin{aligned} \lambda ^5_a(X) = {\left\{ \begin{array}{ll} \displaystyle {\frac{\displaystyle {\sum _{i \in N:\Vert x_{i \cdot } \Vert _1\ge 1}1}}{\Vert X\Vert _1}} &{} \text {if } \Vert X\Vert _1 \ge 1, \\ [0.3cm] 0 &{} \text {otherwise}. \end{array}\right. } \end{aligned}$$
vii) The following index satisfies irrelevance and incidence composition, but it violates neutrality. For each $X \in \mathcal {D}^N$ and each $a \in P$,
  $$\begin{aligned} \lambda ^6_a(X) = {\left\{ \begin{array}{ll} \displaystyle {\frac{1}{\Vert X\Vert _1}\sum _{i=1}^n x_{ia}} &{} \text {if } a=1 \text { and } \Vert X\Vert _1\ge 1, \\ [0.3cm] 0 &{} \text {otherwise}. \end{array}\right. } \end{aligned}$$

$$\square $$

Finally, in the next theorem we establish which properties are satisfied by the four lethality influence indices introduced in Sect. 3. Table 1 shows the properties satisfied for each of these rules.

### Theorem 1

The following statements hold:

i) The count index satisfies neutrality, irrelevance, additive composition, but not sized composition and incidence composition.
ii) The share index satisfies neutrality, irrelevance, sized composition, but not additive composition and incidence composition.
iii) The ratio index satisfies neutrality, irrelevance, incidence composition, but not additive composition and sized composition.
iv) The equal attribution index satisfies neutrality, irrelevance, additive composition, but not sized composition and incidence composition.

### Proof

By using the definitions of the indices is straightforward to prove that they all satisfy *neutrality* and *irrelevance*. Next, we prove what composition property they satisfy and which do not.

1. It is immediate to prove that the count index satisfies additive composition. Now, by Proposition 1 the count index does not satisfy sized composition and incidence composition.
2. Let $N,M \in \mathcal {N}$ such that $N \cap M = \varnothing$. For each $X \in \mathcal {D}^N$ and each $Y \in \mathcal {D}^M$, we have that
  $$\begin{aligned} \begin{array}{ll} \lambda ^S_a(X\oplus Y) &{} = \frac{1}{n+m}\left( \sum _{i=1}^n x_{ia} +\sum _{i=1}^m y_{ia}\right) =\frac{n}{n+m}\frac{1}{n}\sum _{i=1}^n x_{ia} +\frac{m}{n+m}\frac{1}{m}\sum _{i=1}^m y_{ia}\\ [0.3cm] &{} = \frac{1}{n+m}\left( n\lambda ^S_a(X) + m\lambda ^S_a(Y)\right) . \end{array} \end{aligned}$$
  Therefore, the share index satisfies sized composition. However, by Proposition 1 it does not satisfy additive composition, and by Proposition 2 it does not satisfy incidence composition.
3. Let $N,M \in \mathcal {N}$ such that $N \cap M = \varnothing$. For each $X \in \mathcal {D}^N$ and each $Y \in \mathcal {D}^M$. We distinguish three cases:
  - If $\Vert X\Vert _1 = 0$ and $\Vert Y\Vert _1 =0$, it is obvious that
    $$\begin{aligned} \Vert X\oplus Y \Vert _1\lambda ^R(X \oplus Y) = \Vert X \Vert _1\lambda ^R(X) + \Vert Y \Vert _1\lambda ^R(Y). \end{aligned}$$
  - If $\Vert X\Vert _1 = 0$ and $\Vert Y\Vert _1 >0$, then
    $$\begin{aligned} \begin{array}{ll} \Vert X\oplus Y \Vert _1\lambda _a^R(X \oplus Y) &{}= \Vert Y \Vert _1\lambda _a^R(X \oplus Y) = \sum _{i=1}^m y_{ia}=\Vert Y \Vert _1\lambda _a^R(Y)\\ [0.3cm] &{} = \Vert X \Vert _1\lambda _a^R(X) + \Vert Y \Vert _1\lambda _a^R(Y). \end{array} \end{aligned}$$
  - If $\Vert X\Vert _1 > 0$ and $\Vert Y\Vert _1 >0$, reasoning similarly as in (4), we obtain that
    $$\begin{aligned} \Vert X\oplus Y \Vert _1\lambda ^R(X \oplus Y) = \Vert X \Vert _1\lambda ^R(X) + \Vert Y \Vert _1\lambda ^R(Y). \end{aligned}$$
  Therefore, the ratio index satisfies incidence composition. However, by Proposition 1 it does not satisfy additive composition, and by Proposition 2 it does not satisfy sized composition.
4. Let $N,M \in \mathcal {N}$ such that $N \cap M = \varnothing$. For each $X \in \mathcal {D}^N$ and each $Y \in \mathcal {D}^M$, since $N \cap M = \varnothing$, $N_a^{*} \cap M_a^{*} = \varnothing$, so we have that
  $$\begin{aligned} \lambda ^E_a(X \oplus Y) = \sum _{i \in N_a^{*}} \frac{x_{ia}}{\Vert x_{i \cdot } \Vert _1} + \sum _{i \in M_a^{*}} \frac{y_{ia}}{\Vert y_{i \cdot } \Vert _1} = \lambda ^E_a(X) +\lambda ^E_a(Y). \end{aligned}$$
  Therefore, the equal attribution index satisfies additive composition. Now, by Proposition 1 it does not satisfy sized composition and incidence composition.

$$\square $$

**Table 1:** Y means that the index fulfills the property and N means that it does not.

	Neutrality	Irrelevance	Additive composition	Sized composition	Incidence composition
Count index	Y	Y	Y	N	N
Share index	Y	Y	N	Y	N
Ratio index	Y	Y	N	N	Y
Equal attribution index	Y	Y	Y	N	N

## Three families of lethality influence indices

In this section, we present our main findings. Theorem 2 and Proposition 4 constitute a theoretical justification for the prominence of the equal attribution index in the measurement of lethality relevance, while Theorem 3 and 4 show characterizations of the families of indices that contain the share index and the ratio index, respectively. We shall observe that the only difference among the three families of indices characterized in this section is the definition of the property of *composition*.

The first result characterizes the family of indices that satisfy neutrality, irrelevance, and additive composition. It states that under these requirements, the relevance must be a weighted sum of the lethality of the individuals.

### Theorem 2

An index $\lambda$ satisfies neutrality, irrelevance, and additive composition if and only if, there exist neutral functions^3^$w_i:\{0,1\}^{P}\rightarrow {\mathbb R}_{\ge 0}$ for each $i\in N$ such that for each $X \in \mathcal {D}^N$ and for each $a \in P$,

$$\begin{aligned} \lambda _a(X) = \sum _{i=1}^n w_i(x_{i\cdot }) x_{ia}. \end{aligned}$$

### Proof

Let $\lambda$ be an index and let $w_i: \{0,1\}^P \longrightarrow \mathbb {R}_{\ge 0}$ be neutral functions such that, for each $X\in {\mathcal D}^{N}$ and for each $a \in P$, $\lambda _a(X) = \sum _{i=1}^n w_i(x_{i\cdot }) x_{ia}$. We start by showing that this index satisfies the three properties.

- Neutrality. Let $X \in \mathcal {D}^N$, and let $\pi$ be a permutation of columns of *X*. For each $a \in P$,
  $$\begin{aligned} \lambda _a(\pi (X)) = \sum _{i=1}^n w_i(\pi (x_{i\cdot })) x_{i\pi ^{-1}(a)} = \sum _{i=1}^n w_i(x_{i\cdot }) x_{i\pi ^{-1}(a)} = \lambda _{\pi ^{-1}(a)}(X). \end{aligned}$$
  Now, when we apply permutation $\pi$ to the vector $\lambda (X)$, its $\pi ^{-1}(a)$-th coordinate becomes the *a*-th coordinate. Therefore, $\lambda (\pi (X))=\pi (\lambda (X))$ holds.
- Irrelevance. Let $X \in \mathcal {D}^N$, and let $a \in P$ such that $x_{ia}=0$ for each $i \in N$.
  $$\begin{aligned} \lambda _a(X) = \sum _{i=1}^n w_i(x_{i\cdot }) \cdot 0 = 0. \end{aligned}$$
- Additive composition. Let $X \in \mathcal {D}^N$ and $Y \in \mathcal {D}^M$. Also, let $Z = X \oplus Y$. For each $a \in P$, we have that
  $$\begin{aligned} \lambda _a(Z) = \sum _{i \in N \cup M} w_i(z_{i\cdot }) z_{ia} = \sum _{i \in N} w_i(x_{i\cdot }) x_{ia} + \sum _{i \in M} w_i(y_{i\cdot }) y_{ia} = \lambda _a(X) + \lambda _a(Y). \end{aligned}$$

Now, we prove the converse. Let $\lambda$ be an index that fulfills *neutrality*, *irrelevance*, and *additive composition*.

Suppose that *N* is a singleton (a group of just one individual $N=\{i\}$). On the one hand, in the application of *irrelevance*, when the pathology *a* is irrelevant we know that $\lambda _a(X) =0$, and the statement holds. On the other hand, due to *neutrality*, $\lambda _b(X)=\lambda _c(X)$ for any pair of non-irrelevant pathologies. Indeed, let $\pi$ be a permutation such that $\pi (b)=c$, $\pi (c)=b$ and $\pi (a)=a$ for all $a \ne b,c$, then we have that,

$$\begin{aligned} \lambda _c(\pi (X)) = \lambda _{\pi ^{-1}(c)}(X) = \lambda _b(X), \end{aligned}$$

but since *b* and *c* are non-irrelevant and *X* is just a vector, $\pi (X)=X$, hence, $\lambda _c(X) = \lambda _b(X)$. Then, we now consider a function $w_i: \{0,1\}^P \longrightarrow \mathbb {R}_{\ge 0}$ such that for each $x_{i\cdot } \in \{0,1\}^P$ with $x_{i\cdot } \ne 0_P$, $w_i(x_{i\cdot })$ is equal to $\lambda _b(x_{i\cdot })$ where *b* is any non-irrelevant pathology in $x_{i\cdot }$, and $w_i(0_P)=0$. Since $\lambda$ satisfies *neutrality*, $w_i$ is a neutral function.

Now, suppose that *N* is such that $n \ge 2$. Notice that

$$\begin{aligned} X = x_{1\cdot } \oplus \cdots \oplus x_{n\cdot }, \end{aligned}$$

where each $x_{i\cdot }$ is a lethality matrix with just one individual. Since $\lambda$ satisfies *additive composition*, it follows that

$$\begin{aligned} \lambda (X) = \lambda (x_{1\cdot }) + \cdots + \lambda (x_{n\cdot }). \end{aligned}$$

Let $a \in P$. We have already seen that $\lambda _a(x_{i\cdot }) = w_i(x_{i\cdot }) x_{ia}$ for any $i \in N$. Therefore,

$$\begin{aligned} \lambda _a(X) = \sum _{i=1}^n w_i(x_{i\cdot }) x_{ia}. \end{aligned}$$

$$\square $$

By inspecting Table 1 we can conclude that the share and ratio indices do not belong to the family characterized in Theorem 2. On the other hand, the count index is an element of the family, with $w_i(x_{i\cdot })=1$, $\forall i \in \mathbb {N}$. The equal attribution index also belongs to this family, with4

Among the many indices that satisfy neutrality, irrelevance, and additive composition, the equal attribution index presents a distinctive particularity. Since the index is closely related to a well-known solution of cooperative games, it can be grounded from a game theoretic perspective. We can naturally define a cooperative game as follows.^5^ The set of players is the set of pathologies *P*, and the value function for each coalition $Q\subset P$ is given by

1 $$\begin{aligned} v(Q) = \left| \left\{ i \in N : x_{ia}=1 \text { for some } a \in Q \right\} \right| , \end{aligned}$$

where |*T*| is the cardinality of set *T*.

For a given cooperative game (*P*, *v*), a solution is a vector $s \in \mathbb {R}^p_{\ge 0}$ such that $\sum _{a \in P} s_a = v(P)$, where $s_i$ represents the allocation to player *i*. Several authors have proposed different solution concepts based on different notions of fairness. Among those, the *Shapley value* (Shapley 1953) emerges as the most prominent (see Roth 1988; Algaba et al. 2019b). Its expression is the following. For each $a \in P$,

$$\begin{aligned} \text {Sh}_a(v)=\sum _{Q \subset P \backslash \{a\}} \gamma (P,Q)(v(Q \cup \{a\})-v(Q)), \end{aligned}$$

where $$\gamma (P,Q) = \frac{|Q| !(p-|Q|-1) !}{p!}$$.

### Example 3

For the lethality matrix of Example 1, the associated value function is

$$\begin{aligned} \begin{array}{llll} v(\varnothing )=0, &{} v(\{1\}) = 3, &{} v(\{1,2\}) = 5, &{} v(\{1,2,3\})=5, \\ &{} v(\{2\}) = 3, &{} v(\{1,3\}) = 5, &{} \\ &{} v(\{3\}) = 4, &{} v(\{2,3\}) = 4. &{} \\ \end{array} \end{aligned}$$

The corresponding Shapley value is

$$\begin{aligned} \text {Sh}_a(v) = \left( \frac{11}{6}, \frac{8}{6}, \frac{11}{6} \right) . \end{aligned}$$

As we can observe, the equal attribution index in Example 2 and the Shapley value in Example 3 provide the same lethality relevance. The next proposition states that they always coincide.

### Proposition 4

The equal attribution index and the Shapley value of the cooperative game with the value function given in (1) coincide.

### Proof

We first prove that the Shapley value satisfies neutrality, irrelevance, and additive composition. It is obvious that the Shapley value satisfies neutrality and irrelevance. Now we prove that the Shapley value also satisfies additive composition. Let $X \in \mathcal {D}^N$, $Y \in \mathcal {D}^M$, and $Z = X \oplus Y \in \mathcal {D}^{N \cup M}$, and let $(P,v^{X}), (P,v^{Y})$ and $(P,v^{Z})$ be the cooperative games associated with *X*, *Y*, and $X \oplus Y$, respectively. For each $a \in P$,

$$\begin{aligned} \text {Sh}_a(v^{Z})&= \sum _{Q \subset P \backslash \{a\}} \gamma (P,Q)\left( v^Z(Q \cup \{a\}) - v^Z(Q)\right) \\&= \sum _{Q \subset P \backslash \{a\}} \gamma (P,Q)\left( v^X(Q \cup \{a\})+v^Y(Q \cup \{a\}) - v^X(Q) - v^Y(Q)\right) \\&=\sum _{Q \subset P \backslash \{a\}} \gamma (P,Q)\left( v^X(Q \cup \{a\}) - v^X(Q)\right) \\&\quad + \sum _{Q \subset P \backslash \{a\}} \gamma (P,Q)\left( v^Y(Q \cup \{a\}) - v^Y(Q)\right) \\&=\text {Sh}_a(v^{X}) + \text {Sh}_a(v^{Y}). \end{aligned}$$

Therefore, by Theorem 2, for each $X \in \mathcal {D}^N, N \in \mathcal {N}$, $\text {Sh}_a(v^X) = \sum _{i=1}^n w_i(x_{i\cdot }) x_{ia}$, for some functions $w_i: \{0,1\}^P \longrightarrow \mathbb {R}_{\ge 0}, i \in \mathbb {N}$.

Let $X \in \mathcal {D}^N$ such that $N=\{i\}$ (i.e., a singleton), and let $(P,v^X)$ be the associated game. We distinguish two cases:

1. $\Vert x_{i \cdot } \Vert _1 \ge 1$. By the definition of the game, $v^X(P) = 1$. Since the Shapley value is efficient (Shapley 1953), it follows that $\sum _{a \in P} \text {Sh}_{a}(v^X) = v^X(P) = 1$. Now we have the following chain of equalities:
  $$\begin{aligned} \sum _{a \in P} \text {Sh}_{a}(v^X) = \sum _{a \in P}w_i(x_{i\cdot }) x_{ia} = w_i(x_{i\cdot }) \sum _{a \in P} x_{ia}= w_i(x_{i\cdot }) \Vert x_{i \cdot } \Vert _1=1. \end{aligned}$$
  Therefore, $$w_i(x_{i\cdot }) = \frac{1}{\Vert x_{i \cdot } \Vert _1}$$. Thus, $$\mathrm {Sh}_a(v^X)=\frac{x_{ia}}{\Vert x_{i\cdot }\Vert _1}=\lambda _a^{E}(X).$$
2. $\Vert x_{i \cdot } \Vert _1 = 0$. By the definition of the game, $v^X(P) = 0$. Now, it is obvious that for all $a \in P$, $\text {Sh}_a(v^X) = 0=\lambda _a^{E}(X)$.

Therefore, the equal attribution index and the Shapley value coincide for singletons.

Let $X \in \mathcal {D}^N$ such that $n \ge 2$. *X* can be written as

$$\begin{aligned} X = x_{1\cdot } \oplus \cdots \oplus x_{n\cdot } \end{aligned}$$

It is known that the equal attribution index and the Shapley value coincide for each of the singletons. Therefore, by *additive composition*, both coincide in general. $\square$

Therefore, we have shown that the justification for using the equal attribution index is twofold: first, it belongs to the family of indices that is uniquely determined by a suitable combination of properties (Theorem 2); and second, it corresponds to the Shapley value of the associated natural game (Proposition 4).

As pointed out above, the share and ratio indices do not belong to the family characterized in Theorem 2. However, the following results identify the families of indices of lethality relevance to which the share and ratio indices belong. Each family is determined by only changing the concept of composition in use. Thus, the share index belongs to the family of indices determined by *neutrality*, *irrelevance*, and *sized composition* together, while the ratio index belongs to the family of indices determined by *neutrality*, *irrelevance*, and *incidence composition* together.^6^

### Theorem 3

An index $\lambda$ satisfies neutrality, irrelevance, and sized composition if and only if, there exist neutral functions $w_i:\{0,1\}^{P}\rightarrow {\mathbb R}_{\ge 0}$ for each $i\in N$ such that for each $X \in \mathcal {D}^N$ and for each $a \in P$,

$$\begin{aligned} \lambda _a(X) = \frac{1}{n}\sum _{i=1}^n w_i(x_{i\cdot }) x_{ia}. \end{aligned}$$

### Proof

Let $\lambda$ be an index and let $w_i: \{0,1\}^P \longrightarrow \mathbb {R}_{\ge 0}$ be neutral functions such that, for each $X\in {\mathcal D}^{N}$ and for each $a \in P$, $\lambda _a(X) =\frac{1}{n} \sum _{i=1}^n w_i(x_{i\cdot }) x_{ia}$. We start by showing that this index satisfies the three properties. The proofs that this index satisfies neutrality and irrelevance are analogous to the corresponding ones in Theorem 2. Regarding sized composition, let $X \in \mathcal {D}^N$ and $Y \in \mathcal {D}^M$. Also, let $Z = X \oplus Y$. For each $a \in P$ we have that

$$\begin{aligned} (n+m)\lambda _a(Z)= & {} (n+m)\frac{1}{n+m} \sum _{i \in N \cup M} w_i(z_{i\cdot }) z_{ia}\\= & {} n \frac{1}{n} \sum _{i \in N} w_i(x_{i\cdot }) x_{ia} +m\frac{1}{m} \sum _{i \in M} w_i(y_{i\cdot }) y_{ia}\\= & {} n \lambda _a(X) +m \lambda _a(Y). \end{aligned}$$

Conversely, let $\lambda$ be an index that fulfills *neutrality*, *irrelevance*, and *sized composition*.

Suppose that *N* is a singleton. By *irrelevance*, when the pathology *a* is irrelevant we know that $\lambda _a(X) =0$, and the statement holds. Due to *neutrality*, $\lambda _b(X)=\lambda _c(X)$ for any pair of non-irrelevant pathologies. Therefore, there must exist $w_i: \{0,1\}^P \longrightarrow \mathbb {R}_{\ge 0}$ such that $\lambda _b(X)=w_i(x_{i\cdot }) x_{ib}$ for any *b* non-irrelevant.

Suppose next that *N* is such that $n \ge 2$. Then, for each $X \in \mathcal {D}^N$, $X = x_{1\cdot } \oplus \cdots \oplus x_{n\cdot }$, where each $x_{i\cdot }$ is a lethality matrix with just one individual. Since $\lambda$ satisfies *sized composition*, it holds that

$$\begin{aligned} \left( \sum _{i \in N} 1\right) \lambda (X) = 1 \cdot \lambda (x_{1\cdot }) + \cdots +1 \cdot \lambda (x_{n\cdot }). \end{aligned}$$

Let $a \in P$. We have already seen that $\lambda _a(x_{i\cdot }) = w_i(x_{i\cdot }) x_{ia}$ for any $i \in N$. Therefore,

$$\begin{aligned} n \lambda _a(X) = \sum _{i=1}^n w_i(x_{i\cdot }) x_{ia}. \end{aligned}$$

$$\square $$

### Theorem 4

An index $\lambda$ satisfies neutrality, irrelevance, and incidence composition if and only if, there exist neutral functions $w_i:\{0,1\}^{P}\rightarrow {\mathbb R}_{\ge 0}$ for each $i\in N$ such that for each $X \in \mathcal {D}^N$ and for each $a \in P$,

$$\begin{aligned} \lambda _a(X)= {\left\{ \begin{array}{ll} \displaystyle {\frac{1}{\Vert X\Vert _1} \sum _{i=1}^n w_i(x_{i\cdot }) x_{ia}} &{} \text {if } \Vert X\Vert _1 > 0, \\ [0.3cm] 0 &{} \text {if } \Vert X\Vert _1 =0. \end{array}\right. } \end{aligned}$$

### Proof

Let $\lambda$ be an index and let $w_i: \{0,1\}^P \longrightarrow \mathbb {R}_{\ge 0}$ be neutral functions such that, for each $X\in {\mathcal D}^{N}$ and for each $a \in P$,

$$\begin{aligned} \lambda _a(X)= {\left\{ \begin{array}{ll} \displaystyle {\frac{1}{\Vert X\Vert _1} \sum _{i=1}^n w_i(x_{i\cdot }) x_{ia}} &{} \text {if } \Vert X\Vert _1 > 0, \\ [0.3cm] 0 &{} \text {if } \Vert X\Vert _1 =0. \end{array}\right. } \end{aligned}$$

We once again start by showing that this index satisfies the three properties. The proofs that this index satisfies neutrality and irrelevance are *mutatis mutandis* analogous to the corresponding ones in Theorem 2. Regarding incidence composition, let $X \in \mathcal {D}^N$ and $Y \in \mathcal {D}^M$. Also, let $Z = X \oplus Y$. We distinguish three cases:

- $\Vert X\Vert _1 = \Vert Y\Vert _1 = 0$. Therefore, it follows that $\Vert X\oplus Y\Vert _1 = 0$. The result now immediately follows since all factors are zero.
- $\Vert X\Vert _1 >0$ and $\Vert Y\Vert _1=0$. In this case, $\Vert X\oplus Y\Vert _1 =\Vert X\Vert _1$. For each $a \in P$, it holds that
  $$\begin{aligned} \Vert X\oplus Y\Vert _1\lambda _a(Z)= & {} \Vert X\oplus Y\Vert \frac{1}{\Vert X\oplus Y\Vert } \sum _{i \in N \cup M} w_i(z_{i\cdot }) z_{ia} \\= & {} \Vert X\Vert _1 \frac{1}{\Vert X\Vert _1} \sum _{i \in N} w_i(x_{i\cdot }) x_{ia} +0 \cdot \sum _{i \in M} w_i(y_{i\cdot }) \cdot 0\\= & {} \Vert X\Vert _1 \lambda _a(X) +0 \cdot 0 \\= & {} \Vert X\Vert _1 \lambda _a(X)+\Vert Y\Vert _1 \lambda _a(Y). \end{aligned}$$
- $\Vert X\Vert _1 >0$, and $\Vert Y\Vert _1>0$. For each $a \in P$, we have that
  $$\begin{aligned} \Vert X\oplus Y\Vert _1\lambda _a(Z)= & {} \Vert X\oplus Y\Vert \frac{1}{\Vert X\oplus Y\Vert } \sum _{i \in N \cup M} w_i(z_{i\cdot }) z_{ia} \\= & {} \Vert X\Vert _1 \frac{1}{\Vert X\Vert _1} \sum _{i \in N} w_i(x_{i\cdot }) x_{ia} +\Vert Y\Vert _1\frac{1}{\Vert Y\Vert _1} \sum _{i \in M} w_i(y_{i\cdot }) y_{ia} \\= & {} \Vert X\Vert _1 \lambda _a(X) +\Vert Y\Vert _1 \lambda _a(Y). \end{aligned}$$

Conversely, let $\lambda$ be an index that fulfills *neutrality*, *irrelevance*, and *incidence composition*.

Suppose that *N* is a singleton. By *irrelevance*, when the pathology *a* is irrelevant we know that $\lambda _a(X) =0$, and the statement holds. By *neutrality*, $\lambda _b(X)=\lambda _c(X)$ for any pair of non-irrelevant pathologies. Therefore, there must exist $w_i: \{0,1\}^P \longrightarrow \mathbb {R}_{\ge 0}$ such that $\lambda _b(X)=w_i(x_{i\cdot }) x_{ib}$ for any *b* non-irrelevant.

Now, suppose that *N* is such that $n \ge 2$. Then, for each $X \in \mathcal {D}^N$, $X = x_{1\cdot } \oplus \cdots \oplus x_{n\cdot }$, where each $x_{i\cdot }$ is a lethality matrix with just one individual. Since $\lambda$ satisfies *incidence composition*, it holds that

$$\begin{aligned} \left( \sum _{i \in N} \Vert x_{i\cdot } \Vert _1\right) \lambda (X) = \Vert x_{1\cdot } \Vert _1 \cdot \lambda (x_{1\cdot }) + \cdots +\Vert x_{n\cdot } \Vert _1 \cdot \lambda (x_{n\cdot }). \end{aligned}$$

If $\sum _{i \in N} \Vert x_{i\cdot } \Vert _1 = 0$, the result follows since $\lambda$ satisfies *irrelevance*. Let us suppose that $\sum _{i \in N} \Vert x_{i\cdot } \Vert _1 >0$, and let $a \in P$. We have already seen that $\lambda _a(x_{i\cdot }) = w_i(x_{i\cdot }) x_{ia}$ for any $i \in N$. Therefore,

$$\begin{aligned} \left( \sum _{i \in N} \Vert x_{i\cdot } \Vert _1\right) \lambda _a(X) = \sum _{i=1}^n \Vert x_{i\cdot } \Vert _1w_i(x_{i\cdot }) x_{ia}. \end{aligned}$$

Note that $\sum _{i \in N} \Vert x_{i\cdot } \Vert _1 = \Vert X\Vert _1$. Now, we consider the following functions:

$$\begin{aligned} w'_i(x_{i\cdot })=\Vert x_{i\cdot } \Vert _1w_i(x_{i\cdot }), \text { for each } i \in N, \end{aligned}$$

which are also neutral. $\square$

At this stage, we have characterized three families of indices that contain simple and intuitive indices. This means that based on the characteristics of the specific problem, it is possible to select the index that is most compatible with the properties relevant to the problem. Also, we can determine whether there is any differentiation between the individuals of the population when considering them in the calculation of the index. For example, $w_i$ can measure the life expectancy of the individual, in this way, the lethality indices would also take into account the impact on the reduction of the life span of the deceased. Moreover, note that, by Proposition 3, Theorems 2, 3, 4 are tight, that is, all the properties in their statements are necessary for the characterization. Finally, note that none rules given in Proposition 3 satisfy the conclusions of the theorems since each one fails to satisfy one of the three properties required in their statements.

## An application to COVID-19 disease

In this section, we illustrate a possible application of the model presented in the previous sections.

The coronavirus disease 2019 (COVID-19) is an infectious disease caused by severe acute respiratory syndrome coronavirus. First identified in December 2019 in Wuhan, China, COVID-19 resulted in an ongoing pandemic with devastating effects. As of February 2021, around 100 million cases have been reported worldwide, affecting more than 180 countries and resulting in around 2.2 million deaths.

Many researchers have analyzed how preexisting health conditions have influenced COVID-19 mortality. One of the first studies was published by *The Novel Coronavirus Pneumonia Emergency Response Epidemiology Team*. It involved a descriptive and exploratory analysis of all cases of COVID-19 diagnosed nationwide in China in February 2020. Among the several aspects analyzed in the study, the authors identified hypertension, cardiovascular disease, diabetes, and chronic respiratory disease as the main sources of comorbidity among patients who died due to COVID-19 (see Table 2).^7^

**Table 2:** Comorbid conditions reported by The Novel Coronavirus Pneumonia Emergency Response Epidemiology Team

Pathology	% of deaths
Hypertension (HYP)	39.7%
Cardiovascular disease (CAR)	22.7%
Diabetes (DIA)	19.7%
Chronic respiratory disease (RES)	7.9%

A relevant question that many publications have examined, primarily from a medical perspective (see, for example, Zhou et al. 2020; Guan et al. 2020; Li et al. 2020; Nogueira et al. 2020; Richardson et al. 2020; Bello-Chavolla et al. 2020; Stoian et al. 2020, among others), relates to the impact of these four diseases on the lethality of COVID-19. Our theoretical framework constitutes a useful tool that provides an answer to the previous inquiry and quantifies the relative relevance of comorbid pathologies in the lethality of COVID-19.

Let *N* be the set of patients who died with diagnosed COVID-19, and let $P=\{\text {HYP}, \text {CAR}, \text {DIA}, \text {RES}\}$, the set of the the four considered pathologies. The lethality matrix *X*, obtained from the microdata, identifies the preexisting diseases (other than COVID-19 infection) (Table 3).

**Table 3:** Each row corresponds to a patient in the database

Patient	HYP	CAR	DIA	RES
1	1	0	0	0
2	0	0	0	0
3	0	1	1	0
4	1	0	1	1
5	0	1	0	0
$\vdots$	$\vdots$	$\vdots$	$\vdots$	$\vdots$

If a patient has died with the pathology in the column, the value is 1, and 0 otherwise

If the microdata are public, computing the equal attribution index is straightforward, and the medical implications of the results are ready to be analyzed. This is easily achievable by a health authority with access to the information. Unfortunately, these microdata are not publicly available. However, we can overcome the lack of data at an individual level using simulations. Although simulation results are not as reliable compared to the use of real data, we believe they are sufficiently valid to illustrate the application of the theoretical model.

We can ignore the specific entries for the lethality matrix *X*, but we know that the sum of the entries in the first column must coincide with the number of patient deaths that occurred with hypertension. The same reasoning applies to the rest of the columns/pathologies. Therefore, we proceed as follows:

1. Let *N* contain 50 individuals whose pathologies are distributed similarly to Table 2.^8^ In particular, suppose that 20 individuals died with hypertension, 11 individuals died with cardiovascular disease, 10 individuals died with diabetes, and 4 individuals died with chronic respiratory disease.
2. Let $\mathcal {X}$ be the set of all possible lethality matrices that are compatible with the distribution of deaths in the previous step, that is, $X \in \mathcal {X}$ if and only if $\sum _{i \in N} x_{ia} = \text {obs}(a)$ for all $a \in P$, where $\text {obs}(a)$ denotes the observations of pathology *a*.
3. Computationally generate all the matrices in $\mathcal {X}$.
4. For each $X \in \mathcal {X}$ obtained in the previous step, compute the equal attribution index $\lambda ^E(X)$.
5. Finally, average across $\mathcal {X}$, obtaining $\overline{\lambda }^E$.

The results are shown in Table 4. The third column, and probably the most interesting one, indicates the relative relevance of each disease in the lethality caused by COVID-19.^9^ From our findings, we conclude that the relative relevance of hypertension is 51.2%. Interestingly, if we restrict ourselves to the four preexisting diseases, only 44% of patients died with hypertension. In this way, the analysis indicates that the impact of hypertension on deaths due to COVID-19 is significantly greater than what might appear at first glance. On the contrary, the other pathologies are less relevant, with 22.2% vs. 25% for cardiovascular disease, 19.7% vs. 22% for diabetes, and 6.9% vs. 9% for chronic respiratory disease.

**Table 4:** Equal attribution index for COVID-19

Pathology	$\overline{\lambda }^E_a$	$\nu ^E_a$
Hypertension	14.91	0.512
Cardiovascular disease	6.48	0.222
Diabetes	5.75	0.197
Chronic respiratory disease	2.00	0.069

## Conclusions

In 2020, the COVID-19 pandemic left the world devastated. Therefore, one of the main objectives of this paper was to identify the preexisting pathologies that may have contributed to COVID-19 mortality. The ability to quantify the relative relevance of comorbidities on the lethality of COVID-19 may be crucial. In this paper, we established a theoretical model with that purpose in mind.

In this context, we presented several alternatives to measure lethality relevance: *the count, share, ratio, and equal attribution indices*. Following an axiomatic methodology, we proposed several properties that emerge as natural requirements in this context: *neutrality*, *irrelevance*, and three different ways of understanding the concept of *composition*, namely, *additive composition*, *sized composition*, and *incidence composition*. By the combination of *neutrality*, *irrelevance*, and one of the composition properties, we characterized three families of indices for measuring the relative relevance of the pathologies in the lethality of a disease. Theorem 2 states that an index satisfies *neutrality*, *irrelevance*, and *additive composition* if and only if it is a weighted combination of the absence or presence of pathologies in the affected individuals. Theorem 3 states that an index satisfies *neutrality*, *irrelevance*, and *sized composition* if and only if it is an average of a weighted combination of the absence or presence of pathologies in the affected individuals. Theorem 3 states that an index satisfies *neutrality*, *irrelevance*, and *incidence composition* if and only if it is a proportion of a weighted combination of the absence or presence of pathologies in the affected individuals.

On the other hand, the equal share index belongs to the family determined in Theorem 2. Also, Proposition 4 shows that this index coincides with the Shapley value of the associated cooperative game. The application of cooperative game theory in our problem seems very natural. Indeed, we may consider that mortality occurs due to a confluence of cooperating pathologies, from which the need follows to determine how to allocate responsibilities for that loss of life. Therefore, it emerges as the most convenient index of lethality relevance of the family. The share index belongs to the family determined in Theorem 3, whereas the ratio index belongs to the family determined in Theorem 4. These two indices are also simple and intuitive, and they are two relevant members of their respective families of indices.

As an illustration, we applied the proposed theoretical model to quantify the relevance of four pathologies on the lethality of COVID-19: hypertension, cardiovascular disease, diabetes, and chronic respiratory disease. We found that the equal attribution index imputed more relative relevance to hypertension compared to the impact suggested in Team (2020).

Although we justify the application of the equal attribution index, other indices introduced in this paper may deserve deeper analysis. The *count index* is one such index. It identifies the lethality relevance of a pathology with the share of deaths in which the pathology is implicated. It seems that this is precisely what is done implicitly in several medical studies, which simply state the percentage of deaths that occurred when the underlying pathology was present.

Finally, the mathematical approach to the attribution problem adopted in this paper can be also applied to other biological or social contexts, including those different from epidemiology, where a similar mathematical structure may be considered.

## Footnotes

1 Since it may eventually occur that none of the individuals in the society *N* dies while suffering from a pathology in *P*, we must also consider the case of *X* being a null matrix.

2 Notice that, since $\pi$ is a bijective mapping, its inverse function $\pi ^{-1}$ exists and is also bijective.

3 A function $f: \{0,1\}^P \longrightarrow \mathbb {R}_{\ge 0}$ is neutral if for every permutation $\pi$ of the coordinates of vectors $x \in \{0,1\}^P$, $f(\pi (x)) = f(x)$, for all $x \in \{0,1\}^P$.

4 Note that when $\Vert x_{i \cdot } \Vert _1 = 0$, any function value is possible. However, we use $w_i(x_{i\cdot })=0$.

5 This approach has been used to analyze similar contexts (Moretti et al. 2007, 2010).

6 As it is shown in Theorem 1, the share index and the ratio index fulfill the properties in Theorem 3 and Theorem 4, respectively.

7 In 32.8% of deaths from COVID-19, the patient did not have a preexisting disease.

8 Larger numbers of individuals make the simulation computationally intractable.

9 The relative relevance is calculated by normalizing $\overline{\lambda }^E$, i.e., for each $a \in P$, $\nu ^E_a = \frac{ \overline{\lambda }^E_a}{\Vert \overline{\lambda }^E\Vert _1}$.
